# Age-Stratified Mortality Impact of Atrial Fibrillation in Elderly NSTEMI Patients

**DOI:** 10.3390/jcdd13010051

**Published:** 2026-01-16

**Authors:** Ersin Doganozu, Pinar Demir Gundogmus, Emrah Aksakal

**Affiliations:** 1 Department of Cardiology, Faculty of Medicine, Akdeniz University, 07058 Antalya, Turkey; 2 Department of Cardiology, Kırıkkale Yüksek İhtisas Hospital, 71300 Kırıkkale, Turkey; 3 Department of Medical Pharmacology, Institute of Health Science, Ataturk University, 25240 Erzurum, Turkey

**Keywords:** atrial fibrillation, elderly, mortality, NSTEMI, one-year outcome, short-term prognosis

## Abstract

Objectives: Non-ST-segment elevation myocardial infarction (NSTEMI) in the elderly is frequently complicated by multiple comorbidities, which influence clinical outcomes. However, the prognostic significance of atrial fibrillation (AF) in this context remains uncertain. This study aimed to evaluate the impact of AF on short- and long-term mortality in elderly patients (≥65 years) with NSTEMI. Methods: This cross-sectional observational study included 474 NSTEMI patients aged 65 years and older. Participants were stratified into four groups based on age (65–74 vs. ≥75 years) and the presence or absence of AF. One-month and one-year all-cause mortality were assessed as the primary and secondary endpoints, respectively. Results: AF was detected in 23 (11.6%) of 199 patients aged 65–74 and in 80 (29.1%) of 275 patients aged ≥75. While one-month mortality did not differ significantly among the four groups (*p* = 0.514), one-year mortality showed a statistically significant difference (*p* < 0.001). Univariate analysis revealed that AF was not predictive of one-month mortality. In multivariate Cox regression analysis, AF, reduced creatinine clearance, and left ventricular ejection fraction <50% were identified as independent predictors of one-year mortality. Conclusion: AF is not associated with short-term mortality in elderly NSTEMI patients; however, it serves as an independent predictor of one-year mortality. These findings highlight the importance of long-term rhythm monitoring and management in this high-risk population.

## 1. Introduction

Non–ST-segment elevation myocardial infarction (NSTEMI) constitutes the majority of acute coronary syndrome presentations in contemporary clinical practice, with recent guidelines highlighting an increasing relative frequency of NSTEMI compared with STEMI [1,2]. Despite substantial advances in early coronary angiography (CAG) and percutaneous coronary intervention (PCI), NSTEMI remains associated with high morbidity and mortality, especially among elderly patients with multiple comorbidities [3,4]. Conditions such as renal dysfunction, hypertension, and diabetes mellitus are well-established factors that contribute to adverse outcomes within this population. Nevertheless, the prognostic significance of atrial fibrillation (AF), a prevalent arrhythmia that often coexists with non-ST-elevation myocardial infarction (NSTEMI), is not yet fully elucidated [5,6,7,8].

AF is one of the most prevalent cardiac rhythm disturbances worldwide and is known to increase the risk of thromboembolism, heart failure, and death [9]. The incidence of both AF and NSTEMI rises sharply with age [10], and their co-occurrence is especially common among patients aged ≥75 years [11,12]. In elderly NSTEMI patients, AF may be pre-existing or newly diagnosed during hospitalization, and its presence further complicates clinical management by altering decisions around anticoagulation and antiplatelet therapy, thereby increasing the potential for both bleeding and ischemic complications [13,14,15].

Previous studies have shown that AF may adversely affect short- and long-term outcomes in patients with acute myocardial infarction, with some reporting up to a 40% increase in mortality risk compared to patients in sinus rhythm [16]. However, most studies have focused on mixed ACS populations or did not stratify by age, leaving a knowledge gap regarding the specific prognostic impact of AF in elderly NSTEMI cohorts [17]. Additionally, whether the impact of AF on mortality differs between early elderly (65–74 years) and very elderly (≥75 years) patients remains unclear. Furthermore, accurate classification of AF subtypes (e.g., paroxysmal, persistent) typically requires prolonged monitoring, which is often unfeasible in retrospective studies [18].

Given these considerations, the present observational study aims to investigate the impact of AF on one-month and one-year all-cause mortality in NSTEMI patients aged ≥65 years. By stratifying the population into 65–74 and ≥75 years subgroups, we aimed to elucidate potential age-dependent differences in the prognostic significance of AF—an issue of growing importance as the population continues to age.

## 2. Methods

This retrospective cohort study included 474 patients aged 65 years and older who were admitted with a diagnosis of non-ST-segment elevation myocardial infarction (NSTEMI) to Erzurum Regional Training and Research Hospital between January 2016 and December 2019. Patients were eligible if they were aged 65 or older, diagnosed with NSTEMI confirmed by elevated cardiac troponin I (cTnI) levels, and underwent coronary angiography (CAG) during hospitalization.

Patients were excluded if they had a history of coronary artery bypass grafting (CABG), were hospitalized due to decompensated heart failure, had a life expectancy of less than one year (e.g., due to malignancy), or presented with elevated cTnI due to non-cardiac causes. In addition, patients with ventricular arrhythmias, complete atrioventricular block, cardiogenic shock, or cardiac arrest at admission were not included. Those whose cardiac rhythm could not be clearly defined—such as those with ectopic atrial rhythm or junctional rhythm—were also excluded. Furthermore, patients who were referred for CABG following angiography and those diagnosed with severe valvular heart disease were not considered eligible for inclusion.

Of the 568 patients initially screened, 96 were excluded based on the aforementioned criteria, resulting in a final study population of 474 patients.

**Figure 1:**
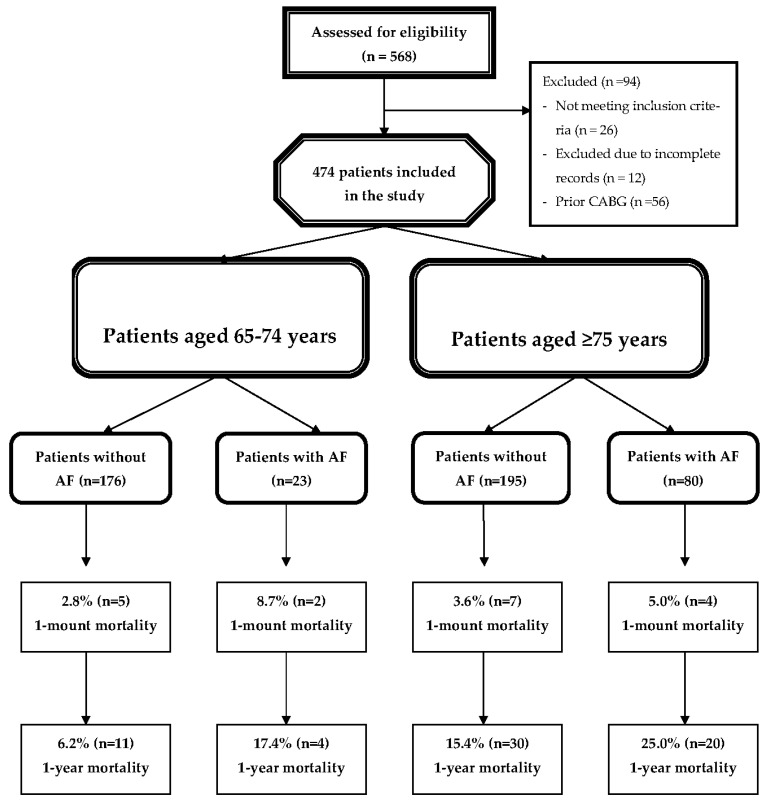
Flow chart of the study. Abbreviations: AF: Atrial Fibrillation; CABG: Coronary Artery Bypass Grafting.

The study was approved by the local ethics committee (IRB: 2020/09-98) and conducted in accordance with the principles outlined in the Declaration of Helsinki. The flowchart of patient selection is shown in Figure 1.

### 2.1. Study Design

Non-ST-segment elevation myocardial infarction (NSTEMI) was defined according to the European Society of Cardiology guidelines, requiring a characteristic rise and/or fall in cardiac troponin levels, ischemic symptoms, and electrocardiographic (ECG) changes indicative of myocardial ischemia. A cardiac troponin I (cTnI) level of 0.1 ng/mL was used as the threshold for myocardial injury. ST-segment deviation was defined as a depression of ≥0.5 mV in two or more contiguous ECG leads.

Atrial fibrillation (AF) was defined as either a known history of AF or a new diagnosis made during hospitalization based on 12-lead ECG findings. Due to the retrospective nature of the study and limitations in clinical follow-up data, AF subtypes—paroxysmal, persistent, or permanent—could not be distinguished. Therefore, any patient with at least one documented episode of AF during hospitalization was classified in the AF group, regardless of recurrence or duration. Left ventricular ejection fraction (LVEF) was measured by the modified Simpson method using a Vivid 7 echocardiography system (GE Medical Systems, Oslo, Norway). Creatinine clearance (CrCl) was calculated using the Cockcroft–Gault formula.

All patients underwent coronary angiography (CAG) as per guideline-directed clinical indications. Coronary lesion severity was assessed using the SYNTAX Score I and II [19], and the Gensini score [20] was calculated by assigning weights to the degree and location of each coronary stenosis. Percutaneous coronary intervention (*PCI*) targeting the culprit lesion was considered evidence of coronary revascularization, regardless of additional interventions.

Global Registry of Acute Coronary Events (GRACE) risk scores [21] were calculated using clinical and laboratory parameters, including age, heart rate, systolic blood pressure, creatinine level, history of heart failure, ST-segment deviation, and elevated cardiac biomarkers. Discharge medications were documented for all patients. The cohort was followed for 12 months. The primary endpoint was one-month all-cause mortality, while the secondary endpoint was one-year all-cause mortality. Patients were divided into four groups based on age (65–74 or ≥75 years) and AF status (with or without AF).

### 2.2. Statistical Analyses

All statistical analyses were performed using SPSS software (version 22.0, IBM Corp., Armonk, NY, USA). Continuous variables were expressed as mean ± standard deviation (SD), and categorical variables as frequencies and percentages. One-way analysis of variance (ANOVA) with Tukey’s post hoc test was used for comparing normally distributed continuous variables among groups, whereas the Kruskal–Wallis test was employed for non-normally distributed variables.

Categorical variables were compared using the Pearson chi-square test or Fisher’s exact test, as appropriate. Variables with a significance level of *p* ≤ 0.05 in univariate Cox regression analysis were included in the multivariate Cox regression model to identify independent predictors of mortality. Survival analyses were performed using the Kaplan–Meier method, and comparisons between groups were evaluated using the log-rank test. A two-tailed *p* value of ≤0.05 was considered statistically significant in all tests.

## 3. Results

A total of 474 NSTEMI patients aged 65 years and above were included in the study. Among the 199 patients aged 65–74 years, 23 (11.6%) had atrial fibrillation (AF), whereas 176 (88.4%) did not. In the group aged 75 years and older (*n* = 275), AF was present in 80 patients (29.1%), while 195 (70.9%) had no AF. The baseline demographic and clinical characteristics of the four study groups are presented in Table 1.

To reduce selection bias, patients with incomplete clinical records or missing mortality data were excluded. Therefore, the final study population of 474 patients consisted only of individuals with complete demographic, clinical, and outcome information.

Significant intergroup differences were observed in age, sex distribution, smoking status, renal failure, diabetes mellitus, as well as history of peripheral artery disease, coronary artery disease, and stroke (all *p* < 0.01). Medication usage at discharge, including acetylsalicylic acid (ASA), clopidogrel, warfarin, novel oral anticoagulants (NOACs), and beta-blockers, also differed significantly between the groups (*p* < 0.001 for all).

Table 2 presents the comparison of clinical characteristics among the study groups. Statistically significant differences were observed in ST segment deviation (*p* < 0.001), coronary revascularization (*p* = 0.002), heart rate (*p* < 0.001), left ventricular ejection fraction (LVEF) (*p* < 0.001), GRACE risk score (*p* < 0.001), Gensini score (*p* = 0.010), admission and peak troponin I levels (*p* = 0.003 and *p* = 0.037, respectively), international normalized ratio (INR) (*p* < 0.001), admission glucose (*p* < 0.001), hemoglobin level (*p* = 0.001), and creatinine clearance (CrCl) (*p* < 0.001).

Regarding treatments administered at discharge, significant differences were noted in the prescription of acetylsalicylic acid (ASA), clopidogrel, warfarin, novel oral anticoagulants (NOACs), renin–angiotensin–aldosterone system (RAAS) blockers, and statins (all *p* < 0.05).

While no significant difference was found in one-month mortality among the four groups (*p* = 0.514), a statistically significant difference was observed in one-year all-cause mortality (*p* < 0.001).

The results of the Cox regression analyses identifying predictors of mortality are summarized in Table 3. In the univariate analysis, creatinine clearance (CrCl) (HR: 0.920; 95% CI: 0.893–0.947; *p* < 0.001), left ventricular ejection fraction (LVEF) < 50% (HR: 26.891; 95% CI: 3.579–202.076; *p* < 0.001), Gensini score (HR: 1.067; 95% CI: 1.038–1.097; *p* < 0.001), and hemoglobin level (HR: 0.673; 95% CI: 0.521–0.868; *p* = 0.002) were identified as predictors of one-month mortality.

Regarding one-year mortality, atrial fibrillation (AF) (HR: 2.386; 95% CI: 1.460–3.901; *p* = 0.001), ST-segment deviation (HR: 1.880; 95% CI: 1.153–3.066; *p* = 0.011), CrCl (HR: 0.956; 95% CI: 0.942–0.971; *p* < 0.001), LVEF < 50% (HR: 3.772; 95% CI: 2.227–6.389; *p* < 0.001), Gensini score (HR: 1.018; 95% CI: 1.002–1.034; *p* = 0.032), and hemoglobin level (HR: 0.769; 95% CI: 0.676–0.875; *p* < 0.001) were significant predictors in univariate analysis.

In the multivariate Cox regression model, AF (HR: 2.040; 95% CI: 1.068–3.894; *p* = 0.031), CrCl (HR: 0.948; 95% CI: 0.922–0.975; *p* < 0.001), and LVEF < 50% (HR: 4.226; 95% CI: 2.084–8.572; *p* < 0.001) remained as independent predictors of one-year mortality. The Cox regression model was statistically significant (−2Log Likelihood: 402.617; χ^2^: 41.747; df: 6; *p* < 0.001).

**Figure 2:**
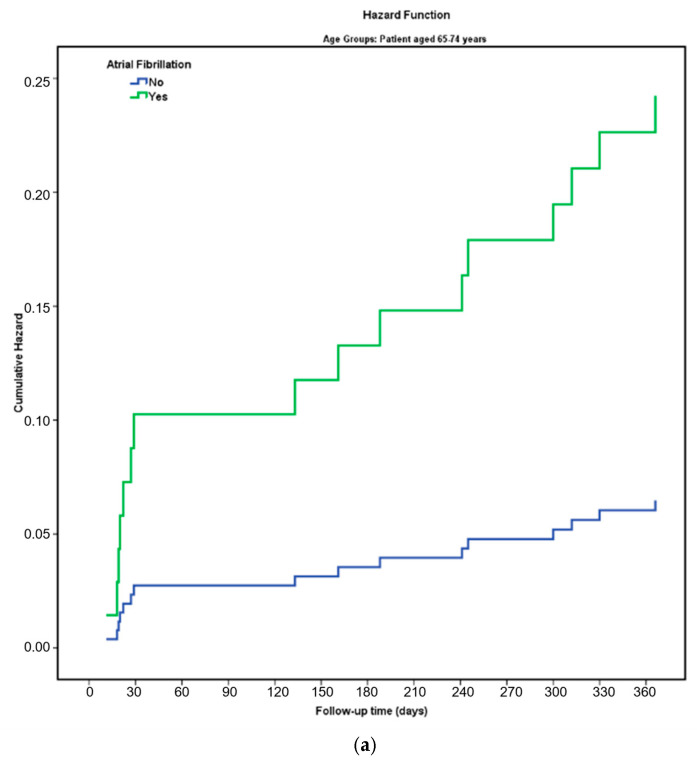

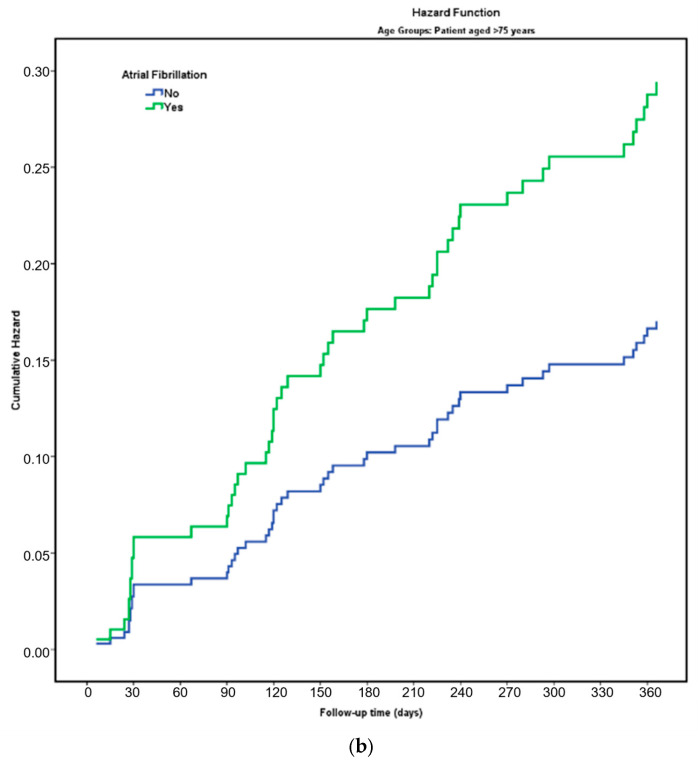
(**a**) The cumulative hazard of overall death in 65–74 years group patients is stratified according to AF. (**b**) The cumulative hazard of overall death in 75 years and over group patients is stratified according to AF. Abbreviations: AF: Atrial Fibrillation.

Figure 2 shows the one-year mortality risk curves by age groups. The Kaplan–Meier survival analysis in Figure 2a showed statistically significant differences between the one-year survival curves of groups 65–74 years old with and without AF (Estimation: 348,744, Std. Err: 5.036, X^2^: 6.910, df:1, Log-Rank *p* = 0.009). The Kaplan–Meier survival analysis in Figure 2b showed no statistically significant differences between the one-year survival curves of groups with and without AF aged > 74 years (Estimation: 327,756, Std. Err: 5.627, X^2^: 3.797, df: 1, Log-Rank *p* = 0.051).

## 4. Discussion

In this cohort of 474 elderly NSTEMI patients (≥65 years), 21.7% had atrial fibrillation (AF). While AF did not significantly influence one-month mortality, it was a strong, independent predictor of one-year all-cause mortality. Additionally, reduced creatinine clearance (CrCl) and left ventricular ejection fraction (LVEF < 50%) emerged as independent predictors of mortality at one year. These findings are notable, as they reflect outcomes in a post-2016 era, during which modern NSTEMI treatments were applied.

AF, already recognized as prevalent in large populations, worsens outcomes when it coexists with acute coronary syndromes (ACS), increasing risks of cardiogenic shock and heart failure [22,23]. Physiologically, AF’s rapid and irregular rhythm reduces diastolic filling and cardiac output. Superimposed on ACS-induced sympathetic activation, this may further impair coronary perfusion and ventricular function. Conversely, impaired LVEF elevates left atrial pressure, perpetuating AF—a self-reinforcing cycle [24]. Furthermore, greater infarct size and systolic dysfunction have been linked to AF onset in AMI patients [6,25].

AF patients typically present with comorbidities—advanced age, hypertension, LV hypertrophy, renal impairment—similar to those in elderly NSTEMI populations [26,27]. We found a remarkably high AF prevalence (≈30%) in patients ≥ 75 years, consistent with expectations and literature data indicating elevated mortality in this demographic.

Management complexity increases in elderly AF patients due to hemorrhagic and ischemic risks, which often lead clinicians to favor oral anticoagulants (OACs) with reduced use of antiplatelets—potentially limiting benefits. Guidelines recommend triple therapy after PCI, but large trials (AUGUSTUS, RE-DUAL PCI) have shown comparable outcomes with dual therapy and reduced bleeding [28,29]. The low rate of antiplatelet use in our AF cohort may have influenced outcomes, although evidence on optimal regimens for elderly patients remains limited.

Revascularization in elderly NSTEMI remains controversial: contemporary meta-analyses show that routine invasive strategies do not significantly reduce one-year all-cause mortality compared with conservative management. However, they may decrease the risk of myocardial infarction and urgent revascularization [30,31,32]. Other studies, however, have reported higher event rates in AF patients undergoing PCI [33,34,35], whereas some have demonstrated a reduction in mortality [36]. Notably, our data showed similar revascularization rates among the groups, yet mortality remained higher in AF patients—a finding suggesting that AF itself portends a worse prognosis, regardless of procedural revascularization.

Previous studies on AMI—including large cohorts and contemporary guideline-level evidence—have established that atrial fibrillation (AF) is associated with increased mortality and adverse cardiovascular outcomes, and that this risk increases with advancing age (e.g., ≥75 years). These studies, however, generally did not stratify elderly patients into subgroups such as early elderly (65–74 years) versus very elderly (≥75 years) when evaluating the prognostic impact of AF in myocardial infarction populations, leaving a gap that our study addresses by detailed age-stratified analysis [37,38].

Additionally, statins—known to reduce stroke and mortality in high-risk patients [39,40]—were used less frequently in the AF subgroup, possibly contributing to the increased mortality observed. ST-segment deviation, an established predictor of NSTEMI complications and death [41,42], was more prevalent in AF patients, aligning with our mortality findings. Finally, our results affirm that lower CrCl and LVEF remain robust indicators of long-term mortality in elderly NSTEMI patients [7,24,25].

Despite guideline recommendations favoring triple or dual antithrombotic therapy in AF patients undergoing PCI, real-world adherence remains suboptimal, particularly in elderly populations. In our cohort, the underuse of antiplatelet agents in the AF group may have contributed to adverse long-term outcomes. This reflects the ongoing clinical dilemma of balancing bleeding versus ischemic risks in this fragile population. Although the causes of death were not determined, it is plausible that AF-related complications, such as thromboembolic events, progressive heart failure, or bleeding from anticoagulant therapy, may have contributed to the increased mortality. Future studies with adjudicated outcomes are needed to clarify the mechanisms underlying AF-related death in NSTEMI patients [43,44].

AF should not be considered merely an isolated arrhythmia but rather a clinical marker of advanced comorbidity and structural heart disease. The higher prevalence of hypertension, diabetes mellitus, reduced renal function, and heart failure in our AF cohort supports this notion. These conditions are known to promote atrial fibrosis and electrical remodeling, thereby increasing AF susceptibility. It is therefore plausible that the excess mortality observed in AF patients may not be solely attributable to the arrhythmia itself but rather to the overall disease burden it represents. In this context, AF may serve as a surrogate marker of cardiovascular frailty and systemic illness in elderly NSTEMI patients [24,26].

### Limitations

This retrospective, single-center study had a limited sample size, especially in age-stratified subgroups, which may affect the statistical power and generalizability. Due to incomplete follow-up data, AF subtypes and causes of death could not be determined. Moreover, key procedural and in-hospital complications were not systematically recorded, which may have influenced outcomes. The lack of such data is a common limitation in retrospective ACS studies and should be considered when interpreting our findings.

In this study, atrial fibrillation (AF) was identified as an independent and significant predictor of one-year mortality in elderly patients with non-ST-segment elevation myocardial infarction (NSTEMI), regardless of age stratification. Additionally, reduced left ventricular ejection fraction (LVEF < 50%) and lower creatinine clearance (CrCl) emerged as robust, independent predictors of long-term mortality. These findings underline the need for closer risk stratification and individualized management strategies in elderly NSTEMI patients, particularly in the presence of AF and impaired renal or ventricular function. The results provide valuable insights into a clinically challenging patient population in which comorbidities frequently limit the effectiveness of guideline-directed therapies. Nevertheless, further large-scale, prospective randomized studies are warranted to confirm our findings and to guide optimal treatment decisions in this high-risk group.

## Figures and Tables

**Table 1:** Baseline Characteristics of The Study Subjects According to existence of AF And Age.

Variable	Patients Aged 65–74 Years [*n* = 199]		Patients Aged ≥75 Years [*n* = 275]		Statistics
	Group 1 Patients with SR [*n* = 176]	Group 2 Patients with AF [*n* = 23]	Group 3 Patients with SR [*n* = 195]	Group 4 Patients with AF [*n* = 80]	
Age [years] [mean ± SD]	70.05 ± 2.71	71.52 ± 2.44	81.02 ± 5.01 *^#^	80.55 ± 4.52 *^#^	*p* < 0.001
Gender [female] [%]	61 [34.7%]	12 [52.2%]	96 [49.2%] *	60 [75.0%] *^#£^	*p* < 0.001
Current Smoke [yes] [%]	40 [22.7%]	1 [4.3%] *	16 [8.2%] *	4 [5.0%] *	*p* < 0.001
COPD [yes] [%]	21 [11.9%]	4 [17.4%]	23 [11.8%]	4 [5.0%] *	*p* = 0.241
RF [yes] [%]	77 [43.8%]	16 [69.6%] *	156 [80.0%] *	72 [90.0%] *^#£^	*p* < 0.001
HT [yes] [%]	119 [67.6%]	19 [82.6%]	141 [72.3%]	60 [75.0%]	*p* = 0.356
DM [yes] [%]	55 [31.2%]	5 [21.7%]	45 [23.1%]	40 [50.0%] *^#£^	*p* < 0.001
Prior PAD [yes] [%]	4 [2.3%]	0	12 [6.2%]	10 [12.5%] *	*p* = 0.006
Prior CAD [yes] [%]	67 [38.1%]	12 [52.2%]	107 [54.9%] *	30 [37.5%] ^£^	*p* = 0.004
Prior PCI [yes] [%]	28 [15.9%]	8 [34.8%]	40 [20.5%]	20 [25.0%]	*p* = 0.103
Prior Stroke [yes] [%]	0	1 [4.3%] *	13 [6.7%] *	4 [5.0%] *	*p* = 0.009
Admission Medications					
ASA [yes] [%]	89 [50.6%]	12 [52.2%]	106 [54.4%]	18 [22.5%] *^#£^	*p* < 0.001
Clopidogrel [yes] [%]	60 [34.1%] ^#^	0	44 [22.6%] *^#^	8 [10.0%] *^£^	*p* < 0.001
Warfarin [yes] [%]	14 [8.0%]	12 [52.2%] *	6 [3.1%] *^#^	16 [20.0%] *^#£^	*p* < 0.001
NOACs [yes] [%]	0	0	6 [3.1%] *	30 [37.5%] *^#£^	*p* < 0.001
RAS blockers [yes] [%]	90 [51.1%]	9 [39.1%]	108 [55.4%]	42 [52.5%]	*p* = 0.488
Beta-Blockers [yes] [%]	99 [56.2%]	17 [73.9%]	85 [43.6%] *^#^	54 [67.5%] ^£^	*p* < 0.001
Calcium Channel Blockers [yes] [%]	66 [37.5%]	10 [43.5%]	62 [31.8%]	20 [25.0%]	*p* = 0.161

Abbreviations: AF: Atrial Fibrillation; ASA: Acetyl Salicylic Acid; CAD: Coronary Artery Disease; COPD: Chronic Obstructive Pulmonary Disease; DM: Diabetes Mellitus; HT: Hypertension; NOACs: Novel Oral Anticoagulants; RAS: Renin–Angiotensin–Aldosterone System; RF: Renal failure; SD: Standard Deviation; SR: Sinus rhythm; PAD: Peripheral Arterial Disease; PCI: Percutaneous Coronary Intervention. *p* < 0.05 was considered statistically significant. * *p* < 0.05, vs. Group 1. ^#^ *p* < 0.05, vs. Group 2. ^£^ *p* < 0.05, vs. Group 3.

**Table 2:** Clinic Characteristics of The Study Subjects According to Age And AF.

Variable	Patients Aged 65–74 Years [*n* = 199]		Patients Aged ≥75 Years [*n* = 275]		Statistics
	Group 1 Patients with SR [*n* = 176]	Group 2 Patients with AF [*n* = 23]	Group 3 Patients with SR [*n* = 195]	Group 4 Patients with AF [*n* = 80]	
ST segment deviation [yes] [%]	39 [22.2%]	11 [47.8%] *	51 [26.2%] ^#^	46 [57.5%] *^£^	*p* < 0.001
Coronary revascularization [yes] [%]	131 [74.4%]	12 [52.2%] *	118 [60.5%] *	42 [52.5%] *	*p* = 0.002
Heart Rate [beat/min] [mean + SD]	81.92 ± 14.21	108.78 ± 21.17 *	79.78 ± 17.85 ^#^	94.07 ± 24.72 *^#£^	*p* < 0.001
Systole Blood Pressure [mm/Hg] [mean + SD]	130.30 ± 14.99	103.60 ± 30.32	133.32 ± 22.95	136.10 ± 20.75	*p* = 0.182
Diastole Blood Pressure [mm/Hg] [mean + SD]	78.25 ± 9.01	75.65 ± 17.00	75.35 ± 13.14	77.80 ± 11.97	*p* = 0.102
LVEF [%] [mean + SD]	51.00 ± 8.99	48.43 ± 12.51	52.14 ± 9.51	46.87 ± 9.69 *^£^	*p* < 0.001
GRACE Score [mean + SD]	123.86 ± 14.20	137.00 ± 22.74 *	140.40 ± 24.51 *	150.67 ± 17.10 *^#£^	*p* < 0.001
Gensini Score [mean + SD]	26.70 ± 15.38	13.26 ± 11.68 *	24.74 ± 18.87	29.97 ± 25.18 ^#^	*p* = 0.010
Admission Troponin [ng/mL] [mean + SD]	2.51 ± 5.84	0.57 ± 0.67	2.41 ± 5.41	0.31 ± 0.54 ^*£^	*p* = 0.003
Peak hs-TnT [ng/mL] [mean + SD]	4.04 ± 8.49	8.61 ± 15.18	4.58 ± 6.94	3.18 ± 5.88 ^#^	*p* = 0.037
INR [mean + SD]	1.21 ± 0.40	1.60 ± 0.85 *	1.13 ± 0.18 ^#^	1.34 ± 0.35 *^#£^	*p* < 0.001
Glucose [mg/dL] [mean + SD]	138.21 ± 70.70	119.65 ± 36.94	130.50 ± 48.92	161.65 ± 59.55 *^#£^	*p* < 0.001
Hemoglobin [g/dL] [mean + SD]	13.19 ± 2.06	12.72 ± 2.55	12.63 ± 1.80 *	12.43 ± 1.76 *	*p* = 0.001
CrCl [mL/dk] [mean + SD]	63.61 ± 21.34	50.96 ± 17.06 *	50.51 ± 12.73 *	45.36 ± 13.59 *	*p* < 0.001
**Hospitalization days [median (IQR)]**	4 (4.75)	6 (7.0)	5 (5.0)	6 (3.0)	*p* < 0.001
Discharge Medications					
ASA [yes] [%]	167 [94.9%]	8 [34.8%] *	188 [96.4%] ^#^	34 [42.5%] *^£^	*p* < 0.001
Clopidogrel [yes] [%]	159 [90.3%]	8 [34.8%] *	172 [88.2%] ^#^	52 [65.0%] *^#£^	*p* < 0.001
Warfarin [yes] [%]	17 [9.7%]	19 [82.6%] *	4 [2.1%] *^#^	28 [35.0%] *^#£^	*p* < 0.001
NOACs [yes] [%]	4 [2.3%]	1 [8.7%]	6 [3.1%]	46 [57.5%] *^#£^	*p* < 0.001
RAS blockers [yes] [%]	120 [68.2%]	11 [47.8%]	139 [71.3%] ^#^	62 [77.5%] ^#^	*p* = 0.047
Beta-Blockers [yes] [%]	152 [86.4%]	19 [82.6%]	175 [89.7%]	72 [90.0%]	*p* = 0.582
Statins [yes] [%]	130 [73.9%]	10 [43.5%] *	131 [67.2%] ^#^	40 [50.0%] *^£^	*p* < 0.001
One-month mortality [yes] [%]	5 [2.8%]	2 [8.7%]	7 [3.6%]	4 [5.0%]	*p* = 0.514
One-year mortality [yes] [%]	11 [6.2%]	5 [21.7%] *	30 [15.4%] *	21 [26,2%] *^£^	*p* < 0.001

Abbreviations: AF: Atrial Fibrillation; ASA: Asetil Salicylic Acid; CrCl: Creatinine Clearance; INR: International Normalized Ratio; hs-TnT: hs-TnT, high-sensitivity troponin T; LVEF: Left Ventricular Ejection Fraction; NOACs: Novel Oral Anticoagulants; RAS: Renin–Angiotensin–Aldosterone System; SR: Sinus rhythm; SD: Standard Deviation. *p* < 0.05 was considered statistically significant. * *p* < 0.05, vs. Group 1. ^#^ *p* < 0.05, vs. Group 2. ^£^ *p* < 0.05, vs. Group 3.

**Table 3:** Predictors of 1-Month and 1-Year Mortality: Cox Regression Analysis.

	Hazard Ratio [95.0% CI]	*p*-Value
One-Month Mortality Univariable Analysis		
AF [yes]	1.820 [0.683–4.851]	0.231
ST segment deviation [yes]	1.812 [0.715–4.592]	0.210
CrCl [One-point increase]	0.920 [0.893–0.947]	<0.001
LVEF < 50% [yes]	26.891 [3.579–202.076]	<0.001
Gensini Score [One-point increase]	1.067 [1.038–1.097]	<0.001
Hemoglobin [One-point increase]	0.673 [0.521–0.868]	0.002
DM [yes]	1.843 [0.727–4.669]	0.197
One-year mortality Univariable analysis		
AF [yes]	2.386 [1.460–3.901]	0.001
ST segment deviation [yes]	1.880 [1.153–3.066]	0.011
CrCl [One-point increase]	0.956 [0.942–0.971]	<0.001
LVEF < 50% [yes]	3.772 [2.227–6.389]	<0.001
Gensini Scores [One-point increase]	1.018 [1.002–1.034	0.032
Hemoglobin [One-point increase]	0.769 [0.676–0.875]	<0.001
DM [yes]	1.272 [0.765–2.114]	0.354
One-year mortality Multivariable analysis		
AF [yes]	2.040 [1.068–3.894]	0.031
ST segment deviation [yes]	0.865 [0.418–1.788]	0.696
CrCl [One-point increase]	0.948 [0.922–0.975]	<0.001
LVEF < 50% [yes]	4.226 [2.084–8.572]	<0.001
Gensini Scores [One-point increase]	0.848 [0.982–1.016]	0.883
Hemoglobin [One-point increase]	0.940 [0.772–1.145]	0.940

Abbreviations: AF: Atrial Fibrillation; CrCl: Creatinine Clearance; DM: Diabetes Mellitus; LVEF: Left Ventricular Ejection Fraction, *p* < 0.05 was considered statistically significant.

## Data Availability

The original contributions presented in this study are included in the article. Further inquiries can be directed to the corresponding author.

## Abbreviations

ACS	Acute Coronary Syndrome
AF	Atrial Fibrillation
AMI	Acute Myocardial Infarction
ASA	Acetylsalicylic Acid
CABG	Coronary Artery Bypass Grafting
CAD	Coronary Artery Disease
cTnI	Cardiac Troponin I
COPD	Chronic Obstructive Pulmonary Disease
CrCl	Creatinine Clearance
DM	Diabetes Mellitus
ECG	Electrocardiogram
GRACE	Global Registry of Acute Coronary Events
hs-TnT	High-Sensitivity Troponin T
HT	Hypertension
INR	International Normalized Ratio
LVEF	Left Ventricular Ejection Fraction
NOACs	Novel Oral Anticoagulants
NSTEMI	Non-ST-Segment Elevation Myocardial Infarction
OAC	Oral Anticoagulants
PAD	Peripheral Arterial Disease
PCI	Percutaneous Coronary Intervention
RAAS	Renin–Angiotensin–Aldosterone System
RF	Renal Failure
SR	Sinus Rhythm
